# Supplementary material for: Knowledge and attitudes of Lebanese women towards Baby Friendly Hospital Initiative practices
Source: PLoS One. 2020 Sep 11;15(9):e0238730. doi: 10.1371/journal.pone.0238730 (PMC7485862; doi:10.1371/journal.pone.0238730)
Supplement: S1 Appendix — (PDF) [file pone.0238730.s001.pdf]

APPENDIX A

Women's Knowledge and perceptions of Baby Friendly Hospital Initiative in Lebanon

Study ID_____________ Date___________________________

Mouhafaza________________________

Age__________________

Gestational Age (completed months):____________

Employment:_______________________________________

Number of children:_________ Number of breastfed children:________

Highest level of education:
Elementary_____ Intermediate_____
Secondary______ University_______

Gross monthly income: < $500_________ $500-1000_______
$1001-5000_____ > $5000_________

1. Do you know what a *Baby Friendly Hospital* is?
   - NO
   - YES

If YES, please explain
____
____
____

If this is your first pregnancy, please go to question 10.

2. In your previous pregnancies, did anyone talk to you about the benefits of breastfeeding?
   - NO
   - YES

If YES, specify who:
____
____

3. In your previous pregnancies, did anyone counsel you about common problems during breastfeeding or about management of breastfeeding when going back to work or home?
   - NO
   - YES

If YES, specify who:

____
____

4. When you delivered your last baby, did you breast feed after birth?
    - NO
    - YES

If YES, how soon after delivery?

____
____

5. When you delivered your last baby, did anyone help you start breastfeeding in the first hour?
    - NO
    - YES

6. When you delivered your last baby, did anyone show you how to breastfeed your baby?
    - NO
    - YES

If YES, explain more:

____
____
____

7. Did anyone inform you how to maintain breastfeeding when you are away from your baby?
    - NO
    - YES
8. What was the first food or drink that your last baby had soon after delivery?
    - Breast milk
    - Formula
    - Dextrose
9. When you delivered you last baby, did the baby stay with you in the same room all the time (24 hours)?
    - YES
    - NO
10. Would you like to deliver in a hospital in which the baby and the mother are kept in the same room from admission till discharge?
    - YES
    - NO

If NO, explain why:

____
____
____

11. Would you deliver your next baby in a hospital that encourages *breastfeeding on demand*, and gives formula only if medically indicated?
    - YES
    - NO

Please explain why:
____
____
____

12. With breastfeeding it is recommended not to give a pacifier to the baby, would you deliver in a hospital that bans pacifiers?
    - YES
    - NO

Please explain why:
____
____
____

13. Do you know about *Skin-to-Skin Contact* between mother and baby?
    - NO
    - YES

If YES, explain more:
____
____
____

14. Did you have any previous experience with skin to skin contact?
    - NO
    - YES

If YES, describe your experience:

- How did you feel:
  ____
  ____
  ____
- Did you feel it helped and how:
  ____
  ____
  ____
- Would you do it again and why: (please explain whether YES or NO)
  ____
  ____
  ____

15. Would you be willing to deliver in a hospital that practices skin to skin contact?
    - YES
    - NO

If NO, please explain why:
____
____
____

16. If the baby was ill and admitted to the hospital for any reason, would you be willing to breastfeed him?
    - YES
    - NO

If NO, please explain why:
____
____
____

17. If you delivered prematurely, would you be willing to pump breastmilk to give to your baby?
    - YES
    - NO

If NO, please explain why:
____
____
____

18. If you delivered prematurely, would you be willing to put the baby to breast if the doctor allows it?
    - YES
    - NO

If NO, please explain why:

Early skin to skin and breastfeeding in babies delivered prematurely improve their health and shorten their stay in the hospital

19. Would you do skin to skin contact?
    - YES
    - NO

If NO, please explain why:
____
____
____

If YEs, for how long at a time and how many times per day?
____
____
____

20. Would you initiate breast feeding or administer pumped milk to your baby early?
    - YES
    - NO

If NO, please explain why:
____
____
____

21. Would you be willing to pump within the first hour after delivery?
    - YES

- NO

If NO, please explain why:
____
____
____

22. In case you could not provide breast milk to your baby, would you be willing to accept breast milk from a donor to be given to your baby? (Assuming that there is a human breastmilk bank where the milk is tested and pasteurized.)
    - YES
    - NO

If NO, please explain why:
____
____
____

23. In your future pregnancy, would you like to communicate with a lactation specialist as soon as you deliver?
    - YES
    - NO

If NO, please explain why:
____
____
____

24. Would you be willing to be referred to a breastfeeding support group after being discharged to help you maintain breastfeeding?
    - YES
    - NO

If NO, please explain why:
____
____
____
