# Supplementary material for: Knowledge and attitudes of Lebanese women towards Baby Friendly Hospital Initiative practices
Source: PLoS One. 2020 Sep 11;15(9):e0238730. doi: 10.1371/journal.pone.0238730 (PMC7485862; doi:10.1371/journal.pone.0238730)
Supplement: S2 Appendix — (PDF) [file pone.0238730.s002.pdf]

إستطلاع بحثي عن آراء ومعرفة ومعتقدات النساء حول المستشفيات الصديقة للطفل في لبنان

استمارة المعلومات

الرقم التسلسلي للدراسة: ______ التاريخ:____________

المحافظة: ____________

العمر : _____ سنة عدد أشهر الحمل:_____ شهر

الوظيفة:__________________________

عدد الأولاد:________

عدد الأولاد الذين تغذوا بالرضاعة الطبيعية:_________

أعلى مستوى علمي حصلت عليه:

□ الابتدائي □ المتوسط □ الثانوي □ الجامعي

المدخول الشّهري:

□ اقل من 500 دولار □ ما بين 500 و 1000 دولار

□ ما بين 1001 و 5000 دولار □ أكثرمن 5000 دولار

1- هل تعرفين ما هي المستشفيات الصديقة للطفل؟ □ كلا □ نعم

إذا كانت الإجابة نعم، الرجاء التوضيح:

____

____

- **إذا كنت حامل للمرة الأولى، الرجاء الذهاب مباشرة إلى السؤال رقم 10**

2- في حمولك السابقة، هل شرح لك أحد من قبل عن فؤائد الرضاعة الطبيعية؟ □ كلا □ نعم

إذا كان الجواب نعم، الرجاء تحديد الشخص الذي قام بذلك:

____

____

3- في حمولك السابقة، هل أخبرك أحد بأكثر المشاكل الحاصلة خلال فترة الإرضاع الطبيعي، وكيف تقومين بالإرضاع الطبيعي عندما تعودين لعملك أو لمنزلك؟ □ كلا □ نعم

إذا كانت إجابتك نعم، الرجاء إخبارنا من هو هذا الشخص:

____

____

4- عند ولادتك لآخر طفل هل قمت بإرضاعه طبيعيا فوراً بعد الولادة؟ □ كلا □ نعم

إذا كانت إجابتك نعم، كم من الوقت بعد الولادة؟ ______________________

5- عند ولادتك لآخر طفل، هل ساعدك أي شخص لبدء الرضاعة الطبيعية بأول ساعة؟

□ كلا □ نعم

6- عند ولادتك لآخر طفل، هل قام أي شخص بتدريبك على كيفية الإرضاع الطبيعي؟

□ كلا □ نعم

إذا أجبت بنعم، الرجاء إخبارنا بالمزيد:

____

____

____

7- هل قام أي شخص بتعليمك كيف تحافظين على الرضاعة الطبيعية عندما تكونين بعيدة عن طفلك؟

□ كلا □ نعم

8- ما كان أول طعام/شراب أخذه طفلك فوراً بعد الولادة؟

- حليب الأم (الثدي)
- الحليب المصنع
- ماء وسكر

9- عندما ولدتي آخر طفل، هل بقي طفلك معك بنفس الغرفة طوال الوقت (24 ساعة)؟

□ كلا □ نعم

10- هل ترغبين أن تلدي في مستشفى تبقي الطفل والأم بنفس الغرفة من وقت الولادة لحين الخروج من المستشفى؟ □ كلا □ نعم

11- هل ترغبين أن تلدي طفلك القادم في مستشفى تشجع الرضاعة الطبيعية الخالصة، وتعطي الحليب المصنع فقط عندما يستوجب ذلك سبب طبي؟ □ كلا □ نعم

الرجاء توضيح السبب:

____

____

____

12- عند الإرضاع الطبيعي، ينصح بعدم إعطاء مصاصة للطفل، هل ستلدين في مستشفى تمنع المصاصات للطفل؟ □ كلا □ نعم

الرجاء توضيح السبب:

____

____

13- هل تعلمين شيئاً عن وضعية اتصال الجلد بالجلد بين الأم والطفل؟

□ كلا □ نعم

إذا كانت إجابتك نعم، الرجاء إخبارنا بالمزيد:

____

____

14- هل لديك تجربة سابقة عن وضعية اتصال الجلد بالجلد؟ □ كلا □ نعم

إذا اجبت بنعم، الرجاء وصف تجربتك:

- بماذا شعرتي____________________________
- هل شعرتي بأنها قد فادتك وكيف؟

____

____

- هل ستقومين بتكرارها ولماذا؟ □ كلا □ نعم

الرجاء التوضيح إذا أجبت بنعم أو لا:

____

____

15- هل ستقومين بالولادة في مستشفى تقوم بممارسة وتطبيق وضعية اتصال الجلد بالجلد؟

□ كلا □ نعم

إذا أجبت ب كلا الرجاء توضيح السبب:

____

____

16- إذا كان طفلك مريضاً ودخل المستشفى لأي سبب، هل ستكملين الإرضاع الطبيعي له؟

□ كلا □ نعم

إذا كان الجواب لا، الرجاء شرح السبب:

____

____

17- إذا ولدت طفل خديج (ولدتي مبكراً) هل ستقومين بسحب حليبك وإعطائه لطفلك؟

□ كلا □ نعم

إذا أجبت بلا، الرجاء توضيح السبب:

____

____

18- إذا ولدت مبكراً، هل ستقومين بوضع طفلك على صدرك مباشرة إذا سمح لك الطبيب بذلك؟
□ كلا □ نعم
إذا أجبت بلا الرجاء توضيح السبب:

____

____

إن ممارسة وضعية إتصال الجلد بالجلد بين الأم والطفل والإرضاع الطبيعي عند الأطفال الخدج يساعد في تحسين صحتهم وتقصير فترة بقائهم في المستشفى

19- هل ستقومين بممارسة وضعية اتصال الجلد بالجلد؟ □ كلا □ نعم
إذا أجبت بلا الرجاء توضيح السبب؟

____

____

إذا أجبت بنعم ما هي المدة وكم مرة باليوم سوف تقومين بها؟

____

____

20- هل ستقومين بالبدء بالإرضاع الطبيعي أم ستمنحين طفلك الحليب المسحوب من ثديك بوقت مبكر؟ □ كلا □ نعم
إذا أجبت بلا الرجاء توضيح السبب:

____

____

21- هل ستقومين بسحب الحليب في اول ساعة بعد الولادة؟ □ كلا □ نعم
إذا أجبت بلا الرجاء توضيح السبب:

____

____

22- في حال عدم قدرتك على تزويد طفلك بحليبك، هل ستقبلين أن يتغذى طفلك بحليب طبيعي من أم متبرعة؟ (*في حال وجود بنك يخزن حليب الثدي البشري ويقوم باختباره وتعقيمه*)
□ كلا □ نعم

إذا كانت إجابتك لا، الرجاء توضيح السبب:

23- في حمولك المستقبلية، هل ترغبين بالتواصل مع خبيرة إرضاع فوراً بعد ولادتك؟

□ كلا □ نعم

إذا أجبت بلا الرجاء توضيح السبب:

23- هل ستقبلين إن يتم إرشادك إلى مجموعة دعم الرضاعة الطبيعية بعد خروجك من المستشفى لمساعدتك على المحافظة على الرضاعة الطبيعية؟ □ كلا □ نعم

إذا أجبت بلا الرجاء توضيح السبب:
