# Supplementary material for: Knowledge and attitudes of Lebanese women towards Baby Friendly Hospital Initiative practices
Source: PLoS One. 2020 Sep 11;15(9):e0238730. doi: 10.1371/journal.pone.0238730 (PMC7485862; doi:10.1371/journal.pone.0238730)
Supplement: Attachment — Submitted filename: comments.docx [file pone.0238730.s004.docx]

Thank you for giving me an opportunity to review this paper. I have some recommendations that are as follows:

1. As the research has done in a Middle East country, I think some articles related to BFHI in other countries should be added. In line 77-78 just mentioned about Canada. How about in Middle East countries?
2. BFHI was lunched by WHO and UNICEF that have ten steps, and step 4 of the Ten steps recommends that “facilitates immediate skin to skin contact”. when this paper aimed to assess the knowledge of women about BFHI steps and skin to skin contact is the 4^th^ step, explore about skin to skin contact separately can be meaningless.
3. What is including criteria? In tables we are not able to see how many women were primipar?
