# Supplementary material for: Knowledge and attitudes of Lebanese women towards Baby Friendly Hospital Initiative practices
Source: PLoS One. 2020 Sep 11;15(9):e0238730. doi: 10.1371/journal.pone.0238730 (PMC7485862; doi:10.1371/journal.pone.0238730)
Supplement: Attachment — Submitted filename: comments (3).docx [file pone.0238730.s006.docx]

Dear editor

Thank you for giving me an opportunity to re-review the paper, all comments have revised in fact author satisfied me.
