# Supplementary material for: Knowledge and attitudes of Lebanese women towards Baby Friendly Hospital Initiative practices
Source: PLoS One. 2020 Sep 11;15(9):e0238730. doi: 10.1371/journal.pone.0238730 (PMC7485862; doi:10.1371/journal.pone.0238730)
Supplement: Attachment — Submitted filename: Response to Reviewers.docx [file pone.0238730.s007.docx]

**DEPARTMENT OF PEDIATRICS AND ADOLESCENT MEDICINE**

**August 20, 2020**

**To: PLOS ONE Editor**

**Subject:** “Knowledge and attitudes of Lebanese women towards Baby Friendly Hospital Initiative practices”

Dear Editor,

We are submitting our revised manuscript PONE-D-20-05601R1 titled, “Knowledge and attitudes of Lebanese women towards Baby Friendly Hospital Initiative practices” for publication in *PLOS ONE*. All changes have been highlighted in yellow in the revised tracked copy. Thank you again for the constructive comments of the reviewers.

**Response to Reviewer #3**

We greatly appreciate your comments and valuable feedback. Please find below our response.

1. Line 186: “Consequently” is probably not the appropriate term. I suggest something like “Once participants had responded to questions xxx the trained research assistant..”.

Answer: Thank you for the suggested edit. We revised the sentence as suggested: “*Once participants had responded to the aforementioned questions, the trained research assistant briefed them about*….”.

Thank you for the thorough review of our paper.

Best wishes.

Yours sincerely,

Mona Nabulsi, MD, MSc

Professor of Clinical Pediatrics

Department of Pediatrics and Adolescent Medicine

Faculty of Medicine

American University of Beirut

Beirut-Lebanon

P.O.Box: 113-6044/C8

Fax:	961-1-370781

961-1-744464

E-mail: [mn04@aub.edu.lb](mailto:mn04@aub.edu.lb)

________________________________________________________________________________________________________

P.O.BOX 11-0236 RIAD EL-SOLH – BEIRUT 1107 2020 – LEBANON – TEL (961 1 374374 – EXT:5500) FAX (961 1 370781 or 744464)

E-MAIL:[peds@aub.edu.lb](mailto:peds@aub.edu.lb)-NEW YORK OFFICE: 850 THIRD AVE.18^TH^ FLOOR–NEW YORK, NY 10022-6297 U.S.A.-TEL. (1 212 583 7677)
